# Bidirectional relationship between the biofilm of *Porphyromonas gingivalis* and the amyloid-beta peptide

**DOI:** 10.1128/spectrum.01981-25

**Published:** 2026-01-16

**Authors:** David Dumoulin, Mnar Ghrayeb, Sarah Côté, Daniel Garneau, Liraz Chai, Eric H. Frost, Tamàs Fülöp, Pascale B. Beauregard

**Affiliations:** 1 Département de Biologie, Université de Sherbrooke 98630 , Sherbrooke, Quebec, Canada; 2 Institute of Chemistry, The Hebrew University of Jerusalem 26742, Jerusalem, Israel; 3 The Harvey M. Krueger Family Center for Nanoscience and Nanotechnology, The Hebrew University of Jerusalem, Jerusalem, Israel; 4 Département de Microbiologie et Infectiologie, Faculté de Médecine et de Sciences de la Santé, Université de Sherbrooke 7321, Sherbrooke, Quebec, Canada; 5 Centre de Recherche sur le vieillissement, Université de Sherbrooke, Sherbrooke, Quebec, Canada; University of the Pacific Arthur A Dugoni School of Dentistry, San Francisco, California, USA

**Keywords:** biofilm, periodontitis, *Porphyromonas gingivalis*, Alzheimer’s disease

## Abstract

Periodontitis and *Porphyromonas gingivalis* infections are significant risk factors for the onset of Alzheimer’s disease (AD). Despite the reliance of *P. gingivalis* on its biofilm for its survival and virulence, the impact of the extracellular matrix on AD’s neuropathological hallmarks has never been examined. In this study, we report a bidirectional relationship between the amyloid-beta (Aβ) peptide, which plays a central role in AD, and the biofilm of *P. gingivalis*. Using multiple fluorescent markers for biofilm components, we observed that Aβ_1-40_ inhibited biofilm formation while Aβ_1-42_ increased extracellular matrix production. Also, using thioflavin T staining and atomic force microscopy, we observed co-aggregation of the biofilm and monomeric Aβ_1-40_, resulting in faster aggregation and significant changes in aggregate structure. Our findings propose mechanistic explanations for the role of *P. gingivalis* as a risk factor for AD and offer potential mechanisms for microbial involvement in AD etiology.

**IMPORTANCE:**

While the etiology of Alzheimer’s disease has been studied extensively for the past 50 years, its exact causes remain unknown. Our current understanding is that the accumulation of multiple genetic and environmental risk factors would lead to the onset of the disease. *Porphyromonas gingivalis* is a bacterium that produces biofilm and elicits periodontitis, a chronic infection of the gums that constitutes a risk factor for Alzheimer’s disease. While studies have looked at the effects of *P. gingivalis* in triggering Alzheimer’s symptoms in animal models, none have explored the impact of the biofilm, which is essential in this bacterium. Our study seeks to bridge that gap by demonstrating a bidirectional relationship between *P. gingivalis* biofilm and amyloid beta, one of the brain lesions involved in Alzheimer’s disease. By understanding the risk factors involved in Alzheimer’s disease and their impact, we aim to provide valuable insights on prevention and treatment.

## INTRODUCTION

The oral biofilm is a complex microbial community that contributes to the onset of multiple diseases, including periodontitis. Periodontitis is a chronic and inflammatory condition of the periodontium characterized by a bacterial-driven degradation of the gum epithelium and the underlying alveolar bone tissue (1). The onset of periodontitis is usually associated with the proliferation of bacteria from the red complex—*Treponema denticola, Tannerella forsythia,* and *Porphyromonas gingivalis—*within the gingival biofilm (2, 3). *P. gingivalis* specifically contributes to the establishment of the oral biofilm by suppressing the host’s immune response and releasing nutrients through periodontal tissue breakdown (4, 5). It also produces gingipains, cysteine proteases targeting lysine-Xaa (Kgp) or arginine-Xaa (RgpA, RgpB) bonds that contribute to intracellular invasion and degradation of antimicrobial peptides (6–10).

Being strictly anaerobic, *P. gingivalis* depends on its biofilm to limit O_2_ exposure. This lifestyle allows for its survival and the secretion of virulence factors. Although *P. gingivalis* biofilm formation is poorly characterized, it relies on the production of two co-occurring fimbriae, the major fimbria FimA and the minor fimbria Mfa1 (11–13). This bacterium also produces capsular polysaccharides that promote interspecies aggregation and extracellular DNA for building its extracellular matrix, but genes encoding these processes are unknown (14, 15). *P. gingivalis* biofilm production also correlates with gingipain secretion, thus contributing to epithelial tissue breakdown, cellular invasion, and release into the bloodstream (16, 17). The progression of multiple diseases such as atherosclerosis (18), rheumatoid arthritis (reviewed in reference 19), and adverse pregnancy outcomes (reviewed in reference 20) is influenced by the propagation of this pathogen. Periodontitis and *P. gingivalis* infections are also confirmed risk factors for Alzheimer’s disease (AD) (21).

AD is an incurable neurodegenerative disease characterized by the accumulation of aggregated amyloid-beta (Aβ) plaques and intracellular neurofibrillary tangles composed of hyperphosphorylated Tau proteins. The occurrence of these lesions triggers neuroinflammation and neuronal cell death, leading to cognitive decline in older adults (22). The toxicity of Aβ is linked to amyloid aggregation into oligomers, which form pores in neuronal cell membranes (23, 24), causing mitochondrial dysfunction (25) and triggering pathogenic inflammation in the central nervous system (CNS) (26). Aβ is mainly secreted in two forms: Aβ_1-40_ represents ~90% of the overall Aβ pool but exhibits low toxicity and aggregation; Aβ_1-42_ is highly aggregative, neurotoxic, and overrepresented in Aβ plaques (27). Outside the CNS, where it is most observed, Aβ is also produced by other cell types, such as skeletal muscle cells, hepatic cells, platelets, and macrophages (28, 29).

The involvement of microbial infection in AD etiology has attracted increasing interest in the last decade (30). Associations between AD and diverse infectious agents revealed that Aβ acts as an antimicrobial peptide against viral, fungal, and bacterial pathogens (31–35). Indeed, in response to different infections, Aβ_1-42_ is produced and accumulates in the CNS (reviewed in reference 32), hinting toward a mechanism in which infections would contribute to AD etiology. Of note, AD development occurs 20–30 years before symptoms appear, which indicates the involvement of a chronic infection rather than an acute event (36).

*P. gingivalis* is one of the most studied pathogens related to AD, as many of its biomolecules, including nucleic acids, lipopolysaccharides, and gingipains, have been observed in various regions of AD brains (reviewed in reference 37). *P. gingivalis* has also been isolated from the cerebrospinal fluid of patients (38, 39). In mouse models, repeated oral infections with *P. gingivalis* resulted in bacterial translocation to the brain, increased Aβ_1-42_ secretion, and AD-like neurodegeneration (40, 41). The interaction between Aβ and *P. gingivalis* also occurs outside the CNS. Notably, Aβ_40/42_ was detected in the gingival crevicular fluid of patients with periodontitis, where it aggregated within the gingival biofilm on the gums (42, 43).

Although *P. gingivalis* is considered a pathogen of interest in AD, the impact of its biofilm on Aβ has not yet been characterized. This multicellular structure is important, as bacterial biofilms have been recovered from Aβ plaques (44–46). A better understanding of the interaction between these two components will provide significant insights into how *P. gingivalis* could be a risk factor for AD and how it can persist despite Aβ’s antimicrobial activity. In this study, we co-cultivated *P. gingivalis* under biofilm-inducing conditions alongside Aβ_1-40_ and Aβ_1-42_ (Aβ_40/42_) and observed that the interaction between Aβ and *P. gingivalis* biofilm varies according to the peptide subtype.

## RESULTS

### Aβ influences *P. gingivalis* biofilm formation

**Fig 1:**
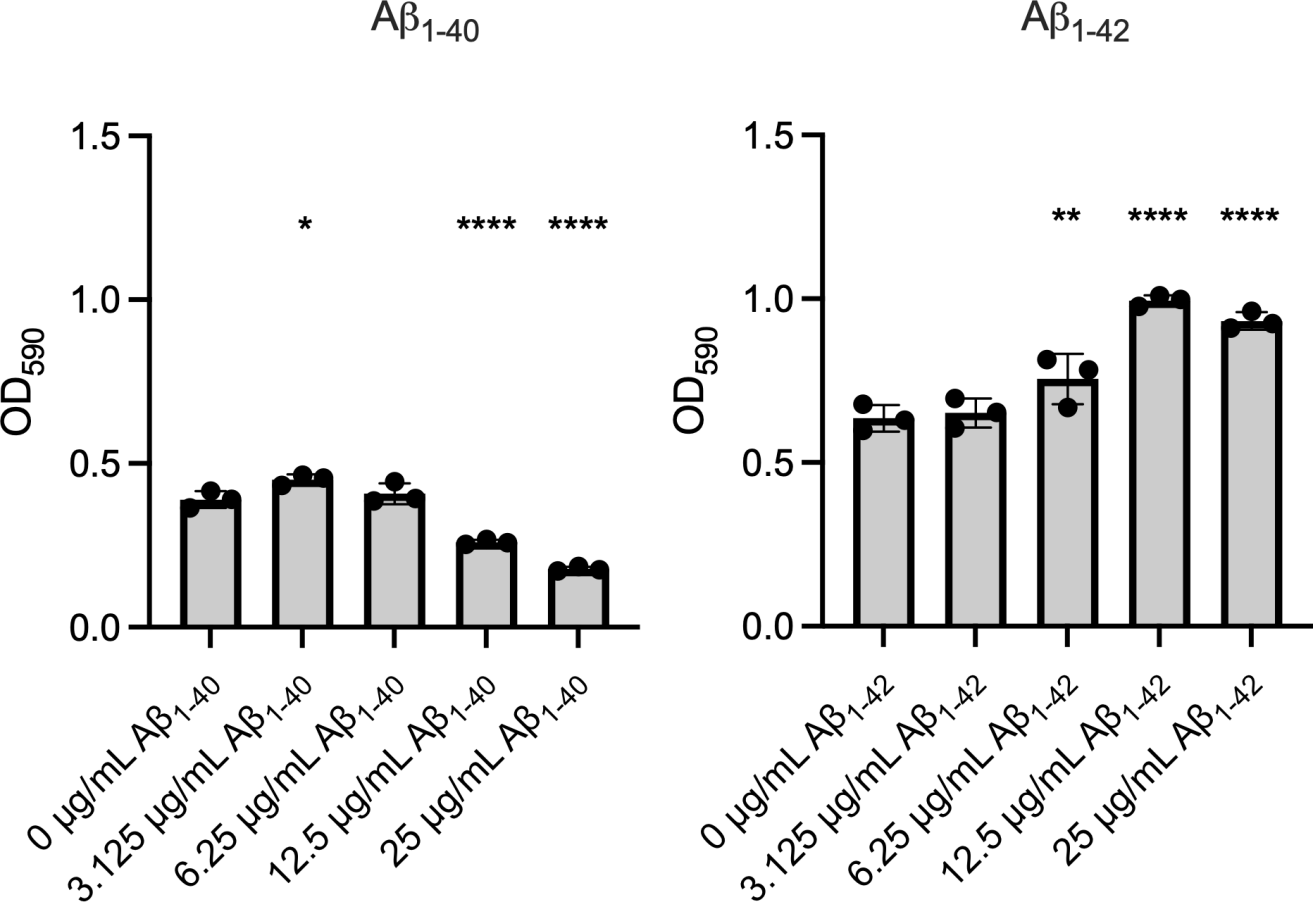
Influence of Aβ_40/42_ on *P. gingivalis* biofilm growth. Biofilm production of *P. gingivalis* in the presence of varying concentrations of Aβ_1-40_ (left) or Aβ_1-42_ (right) in μg/mL, as measured by crystal violet staining after 24 h. This figure contains three technical replicates and is representative of three biological replicates. Statistical analysis was performed using an ANOVA with a Dunnett’s multiple comparison test; * (*P* ≤ 0.05), ** (*P* ≤ 0.005), and **** (*P* ≤ 0.0001) indicate a significant difference from the control (0 μg/mL). Error bars represent the standard deviation (SD).

Previous reports have identified Aβ_40/42_ as an antimicrobial peptide against multiple bacterial species, such as *Escherichia coli*, *Enterococcus faecalis*, and *Staphylococcus epidermidis* (31). To determine if Aβ_40/42_ would impact *P. gingivalis* strain 33277 viability or biofilm formation, *P. gingivalis* was co-incubated for 24 h with various concentrations of Aβ_40/42_ under biofilm-inducing conditions. These Aβ concentrations were chosen to reflect a microenvironment in which bacteria are not abundant but Aβ_40/42_ is, such as in the inflamed part of the brain. Similar concentrations were also used in previous studies (31, 35). After the co-incubation, we quantified biofilm formation using a crystal violet assay. Surprisingly, the two Aβ peptides had different impacts on biofilm formation. High concentrations of Aβ_1-40_ lowered biofilm production at 25 (55% decrease) and 12.5 μg/mL (34% decrease) (Fig. 1). Meanwhile, incubation with Aβ_1-42_ had the opposite effect, resulting in a dose-dependent, significant increase in biofilm formation, peaking between 12.5 (56% increase) and 25 μg/mL (46% increase) (Fig. 1). In parallel, we examined whether Aβ_40/42_ affected *P. gingivalis* viability in planktonic cultures using LIVE/DEAD assays followed by flow cytometry analysis and colony-forming unit (CFU) quantification. Interestingly, the highest concentration of Aβ_1-40_ or Aβ_1-42_ did not affect *P. gingivalis* (Fig. S1).

**Fig 2:**
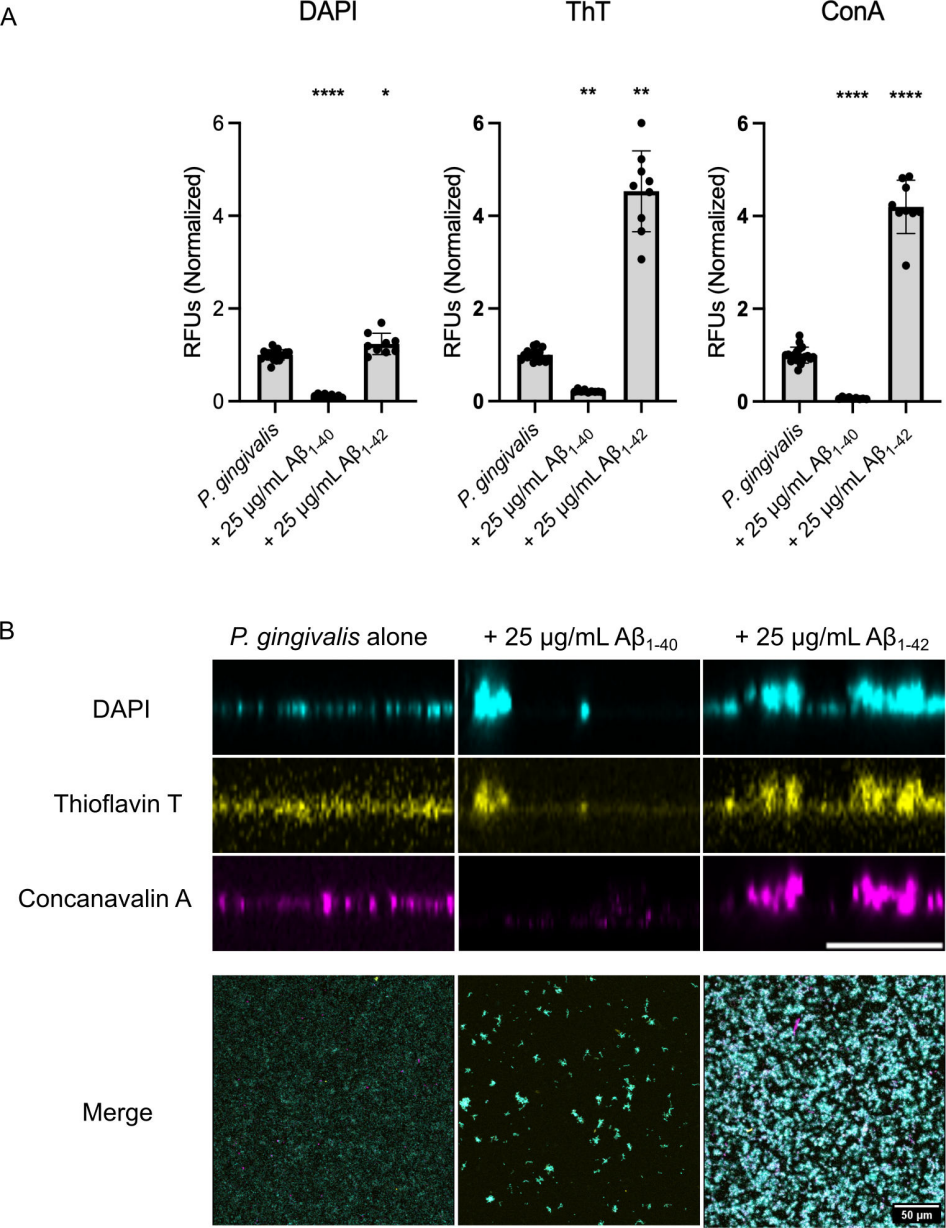
Influence of Aβ_40/42_ on *P. gingivalis* biofilm composition and thickness. (**A**) Fluorescence intensity fold change of DAPI, ThT, and ConA staining on *P. gingivalis* biofilm cultivated with 25 μg/mL Aβ_40/42_ for 24 h. Values for the control were pooled from the individual assays on Aβ_1-40_ and Aβ_1-42_ and normalized against the mean. Statistical analysis was performed using Brown-Forsyth and Welch ANOVA with a Dunnett’s T3 multiple comparison test for DAPI and ConA and Kruskal-Wallis with a Dunn’s multiple comparisons test for ThT, * (*P ≤* 0.05), ** (*P ≤* 0.005), and **** (*P* ≤ 0.0001) indicate a significant difference from the control. Error bars represent SD. (**B**) Cross-sections of *P. gingivalis* biofilm cultivated with 25 μg/mL Aβ_40/42_ for 24 h and visualized with DAPI, ThT, and ConA. Scale bar = 10 μm. Accompanied by the merge of the three channels in a front view. A total of at least 25 images were taken (all biological replicates together). Panel A was generated from a single biological replicate and is representative of all replicates. Scale bar = 50 μm.

Since crystal violet is a non-specific dye, the impact of Aβ_40/42_ on biofilm formation could be due to changes in the amount of extracellular matrix or bacteria within the biofilm. To discriminate between these two possibilities, we used differential fluorescence staining to quantify the abundance of each component. We used the DNA-binding fluorescent molecule 4′,6-diamidino-2-phenylindole (DAPI) to mark the cells. Thioflavin T (ThT) was used as a general protein marker, as a proxy for proteins within the extracellular matrix (47). Despite being quite specific to amyloid fibers, ThT was shown to be fluorescent in the *P. gingivalis* biofilm and in cell-free biofilm fragments (Fig. S2). Finally, the matrix exopolysaccharides were labeled using fluorophore-bound lectin concanavalin A (ConA) (48). These markers were used on *P. gingivalis* biofilms cultivated with 25 μg/mL of Aβ_1-40_ or Aβ_1-42_, and fluorescence was quantified to determine whether cell, protein, or exopolysaccharide levels varied during biofilm formation. For Aβ_1-40_, we observed a significant decrease in the fluorescence intensity of each marker compared to the control with no Aβ (Fig. 2A). Indeed, direct observation of the biofilm by confocal imaging (Fig. 2B) confirmed that in the presence of Aβ_1-40_, the biofilm of *P. gingivalis* was very sparse and organized in small aggregates.

Meanwhile, biofilms of *P. gingivalis* formed with Aβ_1-42_ exhibited a notable increase for each marker compared to the control, the degree of which varied among markers. While the rise in intensity associated with DAPI (~1.25-fold) was significant, it was much lower than the fluorescence increases associated with ThT (~4.5-fold) and ConA (~4.2-fold). This result suggests that the effect of Aβ_1-42_ on the biofilm was due to a strong stimulation of the production and secretion of the extracellular matrix (Fig. 2A). Of note, the sharp increase in the ThT fluorescence intensity in the presence of Aβ_1-42_, which surpasses the increase observed with ConA, could be partly due to ThT binding to the aggregated Aβ within the biofilm. Confocal imaging of biofilm cross-sections showed a thicker community when the biofilm was formed in the presence of Aβ_1-42_ (Fig. 2B), resulting in a higher signal in all channels and a visibly increased biomass.

### Aβ aggregates within the *P. gingivalis* biofilm

**Fig 3:**
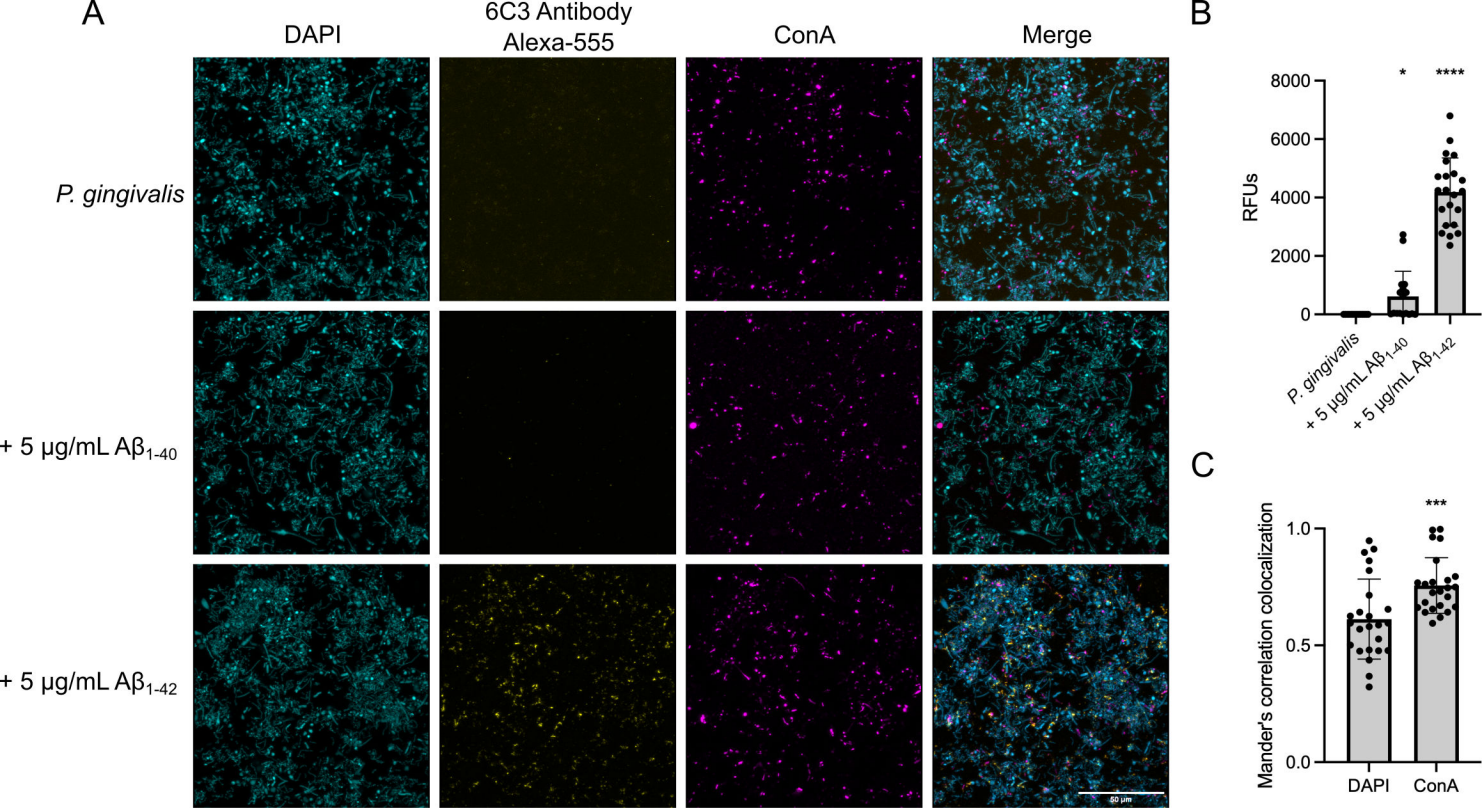
Presence of Aβ_40/42_ within the biofilm of *P. gingivalis*. (**A**) Confocal imaging of Aβ_1-40_ or Aβ_1-42_ (yellow; 6C3 antibody) within the biofilm of *P. gingivalis* after 24 h. Cells are in blue (DAPI) and exopolysaccharides are in magenta (ConA). (**B**) Quantification of the fluorescence of Aβ_1-40_ or Aβ_1-42_ aggregated within the biofilm of *P. gingivalis*. Statistical analysis was performed using the Kruskal-Wallis test with a Dunn’s multiple comparisons test; * (*P ≤* 0.05) and **** (*P* ≤ 0.0001) indicate a significant difference from the control. (**C**) Mander’s colocalization correlation of Aβ_1-42_ with DAPI (cells) and ConA (biofilm). Statistical analysis was performed using the Mann-Whitney test. *** (*P* ≤ 0.001). At least 25 images per condition are included in the analysis, covering three biological replicates.

Previous studies identified biofilm components in the brain of AD patients, interwoven with Aβ plaques (44, 45). We hypothesized that, similarly, Aβ aggregates could be embedded in the biofilm of *P. gingivalis*. Therefore, we imaged biofilms of *P. gingivalis* formed in the presence of 5 μg/mL of Aβ_40/42_. This concentration had only a small effect on biofilm formation (see Fig. S3), which was important because our goal was to determine whether Aβ_40/42_ could be embedded in the biofilm. Matrix components were stained as described in Fig. 2, and Aβ_40/42_ was detected using the MOAB-2/6C3 monoclonal antibody coupled to the Alexa Fluor 555 fluorophore. Interestingly, confocal imaging revealed that Aβ aggregates were present as inclusions in the extracellular matrix of *P. gingivalis* after 24 h. Aβ_1-42_ was more abundant within the *P. gingivalis* biofilm than Aβ_1-40_, which was present only in a few instances (Fig. 3A). Quantification of the Alexa Fluor 555 in the biofilm confirmed that the accumulation of Aβ_1-42_ was significantly higher than Aβ_1-40_ (Fig. 3B). We then assessed if Aβ_1-42_ aggregation was more closely associated with cells (DAPI) or the extracellular matrix (ConA). Mander’s co-localization correlation analysis showed that the signal for Aβ_1-42_ co-localized significantly more with the exopolysaccharides (ConA) channel than with the DAPI labeled cells (Fig. 3C).

### *P. gingivalis* biofilm influences Aβ aggregation

**Fig 4:**
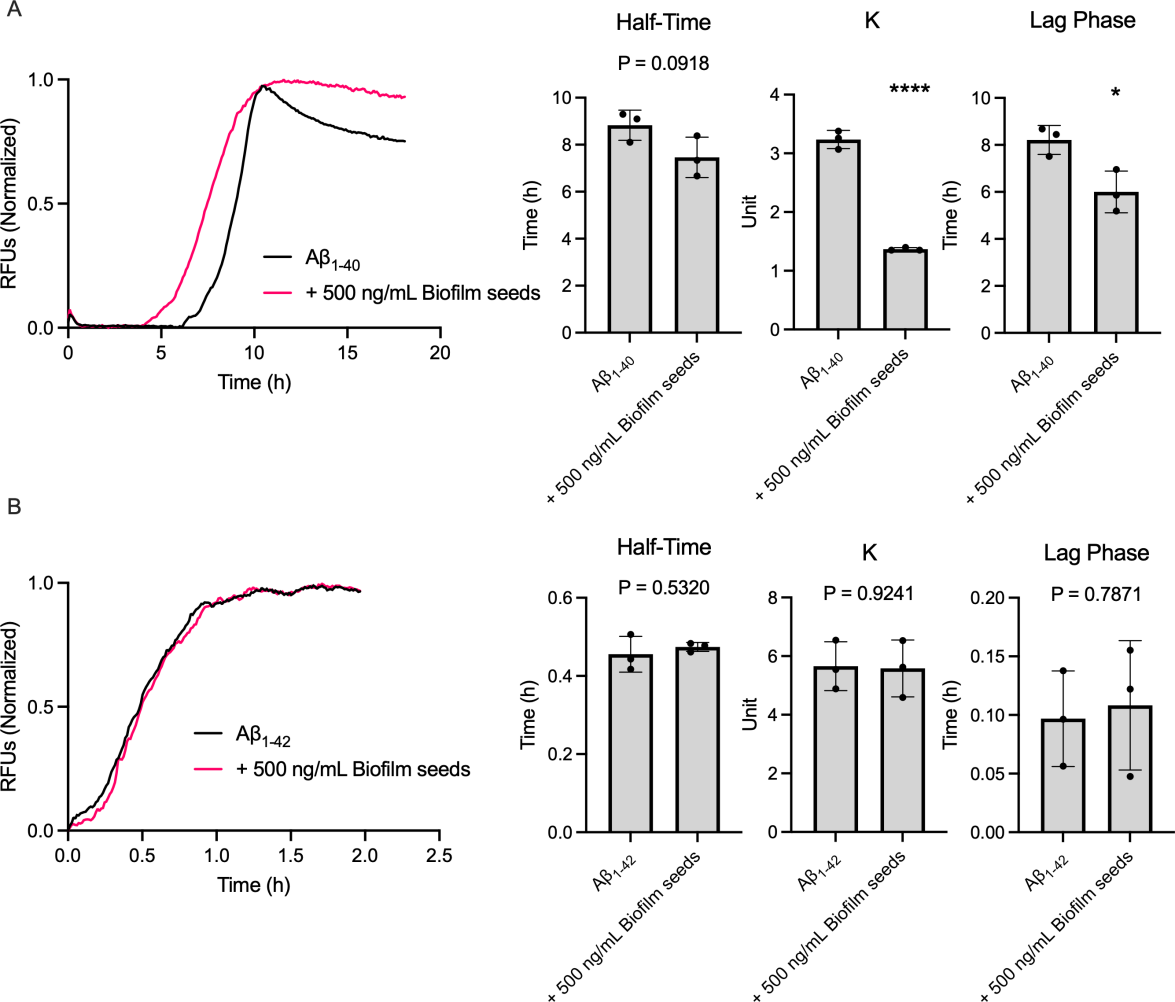
Aggregation kinetics of monomeric Aβ_1-40_ (**A**) and Aβ_1-42_ (**B**) incubated with biofilm seeds from *P. gingivalis*. Half-time (left), aggregation constant (K, middle), and lag phase (right) were extracted from individual curves. Statistical analysis was performed with Student’s *t*-test; * (*P ≤* 0.05), **** (*P* ≤ 0.0001). Error bars represent SD. Curves are the average of three technical replicates and are representative of three biological replicates.

Since Aβ aggregates were visible within the biofilm of *P. gingivalis,* we hypothesized that some biofilm components could interact with Aβ peptide and influence its aggregation rate. We first separated the extracellular biofilm matrix from the bacterial cells to produce nanometric biofilm fragments (hereafter, seeds). These seeds were then incubated with monomeric Aβ_40/42_ in a 1:20 ratio in the presence of ThT, which binds to β-fold structures formed during amyloid aggregation. This binding leads to increased ThT fluorescence, enabling real-time observation of Aβ aggregation. Real-time fluorescence curves were then analyzed by assessing the aggregation constant (k), the aggregation half-time, and the length of the lag phase. For Aβ_1-40_, we observed a significant decrease in lag phase when the peptide was incubated with seeds, as evidenced by the resulting aggregation curve (Fig. 4A). We also observed a non-significant, but still sizable, decrease in half-time. Interestingly, incubation of monomeric Aβ_1-40_ with seeds also reduced the peptide’s aggregation constant by threefold. These results indicate that although the presence of seeds reduced the time necessary for fibrillation of Aβ_1-40_, the actual aggregation rate was slower. Inversely, incubation with seeds had no impact on the aggregation onset or rate for Aβ_1-42_ (Fig. 4B).

**Fig 5:**
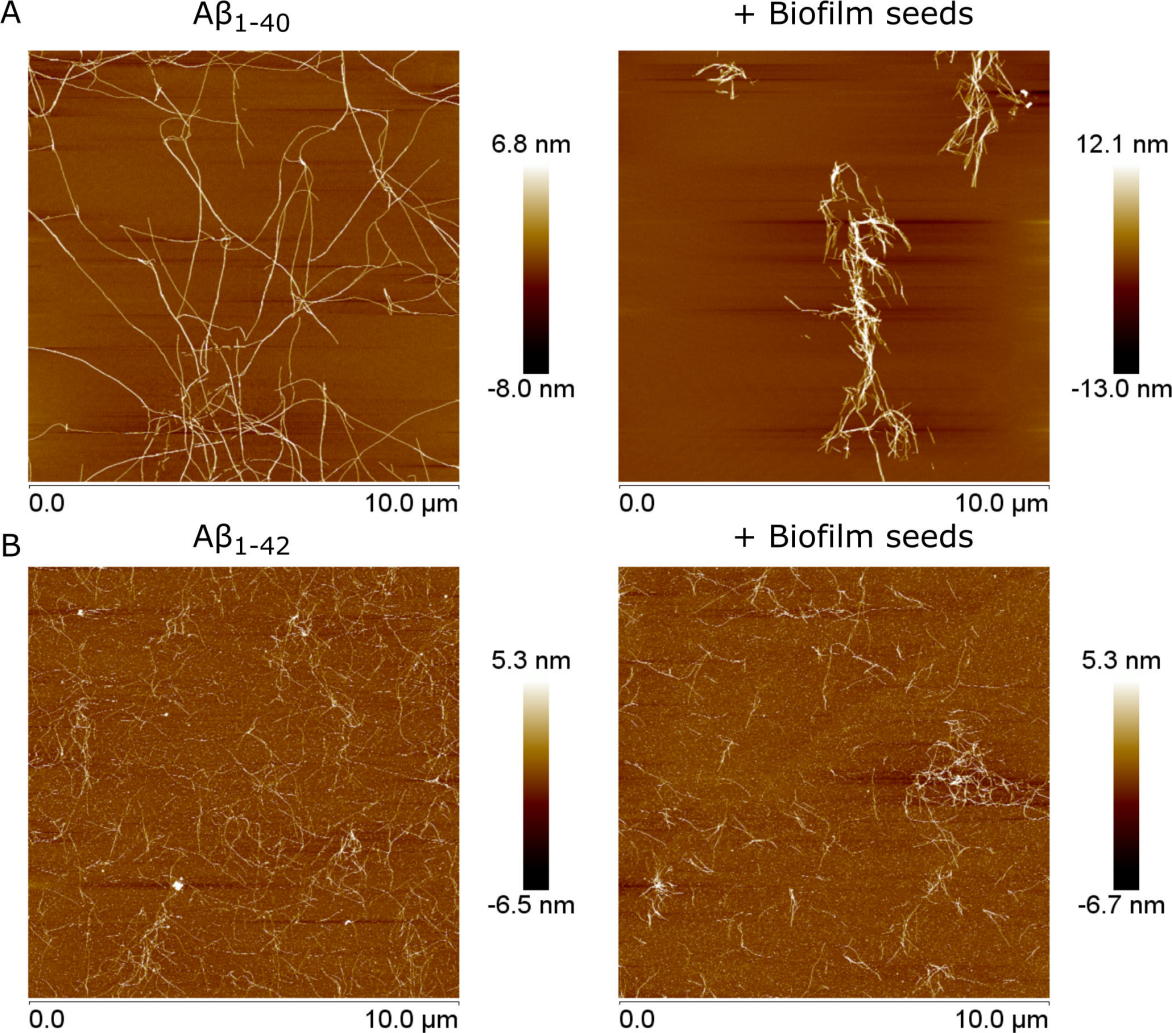
Aggregation of Aβ_1-40_ (**A**) or Aβ_1-42_ (**B**) incubated alone or with biofilm seeds from *P. gingivalis* at 37°C for 3 h (Aβ_1-40_) and 1 h (Aβ_1-42_) and visualized through AFM using height sensors. Images represent 12 technical replicates across three biological replicates.

We then used atomic force microscopy (AFM) to assess whether the increase in Aβ_1-40_ aggregation in the presence of seeds altered the overall structure of fibrils. Briefly, a 100 μg/mL solution of Aβ40/42 was incubated with 5 μg proteins/mL of biofilm seeds for 3 h for Aβ_1-40_ or 1 h for Aβ_1-42_ before being placed on a clean mica surface. As shown in Fig. 5, Aβ_1-40_ alone formed lengthy, complex networks of interconnected fibrils (Fig. 5A). However, in the presence of seeds, Aβ_1-40_ formed tight and isolated bundles of thick fibrils. This seemingly higher aggregative state might explain the decrease in the lag phase necessary for Aβ aggregation observed in Fig. 4A. For Aβ_1-42_, the aggregation pattern between the peptide alone and with seeds showed no significant difference, consistent with results from the ThT aggregation kinetics assays (Fig. 5B).

### Gingipains can break down Aβ

**Fig 6:**
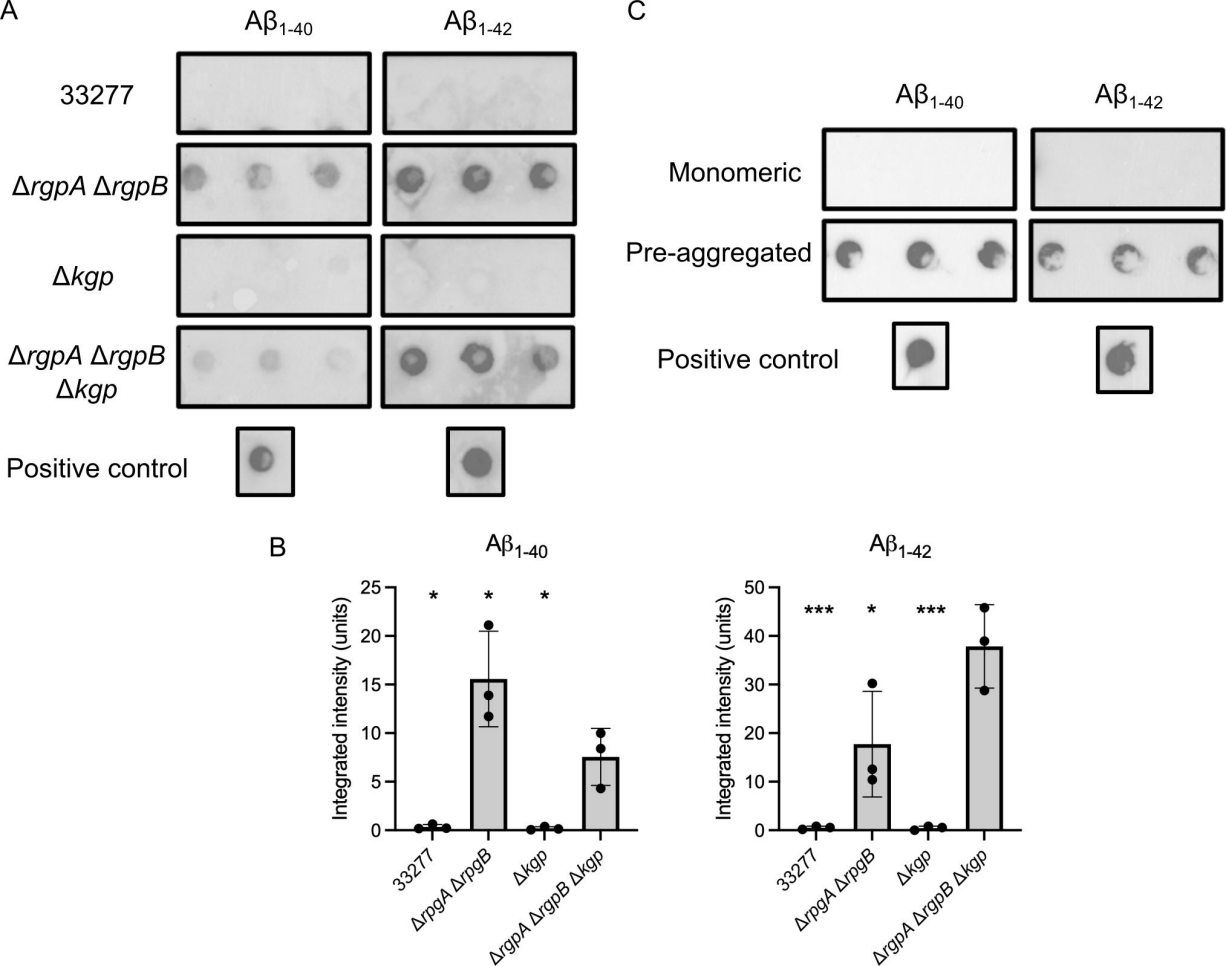
Degradation of Aβ_40/42_ by gingipains from *P. gingivalis*. (**A**) Monomeric Aβ_1-40_ or Aβ_1-42_ incubated with supernatant from *P. gingivalis* WT ATCC 33277, Δ*kgp*, Δ*rgpA*Δ*rgpB*, and Δ*rgpA*Δ*rgpB*Δ*kgp*. (**B**) Densitometry analysis of **A**. Statistical analysis was performed using ANOVA with a Dunnett’s multiple comparison test; * (*P ≤* 0.05) and *** (*P ≤* 0.001) represent a significant difference compared to Δ*rgpA*Δ*rgpB*Δ*kgp*. Error bars represent SD. (**C**) Monomeric and pre-aggregated (24 h at 37°C) Aβ_1-40_ or Aβ_1-42_ incubated with supernatant from *P. gingivalis*. Results are representative of three biological replicates. Positive controls represent Aβ without *P. gingivalis* supernatant.

*P. gingivalis* produces gingipains, large-spectrum proteases able to break down antimicrobial peptides (49). Since *P. gingivalis* biofilm was resistant to Aβ_1-42_ antibacterial properties, gingipains, which are abundant in the biofilm, could be involved in limiting its toxicity. We therefore assessed the proteolytic activity of gingipains on Aβ_40/42_. We first produced gingipain-rich stationary phase supernatants from a 48 h culture of *P. gingivalis* 33277, of the deletion mutants Δ*rgp*AΔ*rgpB* (KDP112), Δ*kgp* (KDP129), and of the triple mutant Δ*rgpA*Δ*rgpB*Δ*kgp* (KDP128). All supernatants were incubated with 5 μg/mL of Aβ_40/42_ for 16 h and then spotted onto PVDF membranes for dot blot assays. As shown in Fig. 6A, both Aβ_1-40_ and Aβ_1-42_ were degraded when incubated with supernatants from *P. gingivalis* 33277, demonstrating that the bacterial supernatant can break down the peptide. Exposure to the supernatant of Δ*rgpA*Δ*rgpB*Δ*kgp* did not affect the peptide, suggesting that gingipains are responsible for Aβ_40/42_ degradation. Interestingly, the supernatant produced by the Δ*rgpA*Δ*rgpB* strain also did not break down Aβ_40/42_, whereas the supernatant from Δ*kgp* did (Fig. 6A). Densitometry analysis (Fig. 6B) revealed a more pronounced degradation of Aβ₁₋₄₀ by the Δ*rgpA*Δ*rgpB* mutant compared to the Δ*rgpA*∆*rgpB*∆*kgp* mutant, which could result from variability in the assay or from the upregulation of *kgp* or another protease in the double arginine-specific gingipains mutant. Interestingly, gingipains could only digest monomeric Aβ_40/42_, while fully aggregated fibrils were unaffected (Fig. 6B). Aβ fibrils are notoriously complex to break down, requiring a multi-step process that includes various proteases (50). In our case, gingipains alone were insufficient to fully degrade pre-assembled Aβ complexes.

## DISCUSSION

While the link between AD and *P. gingivalis* has been extensively investigated over the past 10 years, no study has examined the specific impact of the biofilm on the AD hallmark, Aβ (21, 39, 41, 51–57). Using a simplified model, we observed a bidirectional relationship specific to each peptide. Indeed, both Aβ_40/42_ impacted *P. gingivalis* biofilm production differently, but the biofilm only influenced Aβ_1-40_ aggregation.

We first determined that Aβ_1-40_ and Aβ_1-42_ had different effects on *P. gingivalis*. Biofilm production was decreased when exposed to increasing concentrations of Aβ_1-40_, while Aβ_1-42_ increased the abundance of the extracellular matrix. These results are surprising considering that Aβ_1-42_ is more toxic than Aβ_1-40_ (58). Importantly, Aβ_1-42_ did not exhibit antimicrobial activity against *P. gingivalis*, since it stimulated biofilm formation and did not affect the viability of planktonic cells. This lack of antagonism might be due to the abundance of proteinases secreted by *P. gingivalis*, as discussed later. Although Aβ_1-42_ was not cross-seeded by the biofilm seeds, its presence still enhanced biofilm production in a dose-dependent manner (Fig. 1 and 2). One hypothesis is that aggregated Aβ_1-42_ clumps could promote bacterial aggregation, thereby stimulating increased matrix secretion. Indeed, the bacterial clumping effect of Aβ has been described in other bacteria (59), most notably in *Salmonella typhimurium* (60)*.*

In contrast to Aβ_1-42_, high concentrations of Aβ_1-40_ reduced biofilm formation. These observations are not unlike those made on *E. coli,* for which the presence of Aβ_1-42_ decreased biofilm production (61, 62). Interestingly, this bacterium relies on the presence of the functional amyloid protein CsgA as part of its extracellular matrix. CsgA can cross-seed Aβ_1-42_ and form unproductive heterogeneous polymers, leading to a disruption of the biofilm (61). While there are currently no known functional amyloid proteins in *P. gingivalis* biofilm, aggregation kinetic assays (Fig. 4) and AFM imaging (Fig. 5) showed that the Aβ_1-40_ aggregation pattern differed in the presence of biofilm seeds. These results suggest the existence of a co-aggregation phenomenon between Aβ_1-40_ and a component of the biofilm matrix. This co-aggregation appears unproductive, as it limits biofilm formation by preventing matrix secretion and assembly (Fig. 2), similar to what was observed with *E. coli* (61).

Aggregation kinetics assays (Fig. 4) and AFM imaging (Fig. 5) with Aβ_1-42_ showed no difference when incubated with *P. gingivalis* biofilm. This result could stem from faster Aβ_1-42_ self-assembly kinetics compared to its potential interaction with a biofilm component. Indeed, Aβ_1-42_ has a high aggregation/oligomerization potential and might favor homogeneous aggregation rather than cross-interactions (reviewed in reference 63), thereby not interfering with *P. gingivalis* biofilm matrix polymerization (Fig. 2).

Aβ_40/42_ inclusions within the biofilm appeared unrelated to co-aggregation capacity, as we observed both Aβ_1-42_ and Aβ_1-40_ embedded within the matrix. These inclusions seemed to result from Aβ_40/42_ aggregates trapped in the extracellular matrix following deposition (64), which would also explain the co-localization with exopolysaccharides. Only a small number of Aβ_1-40_ inclusions were observed compared to Aβ_1-42_, which might be due to the slower aggregation rate of Aβ_1-40_ and the presence of gingipains produced by *P. gingivalis*. Indeed, gingipains can degrade monomeric, but not pre-aggregated Aβ (Fig. 6), since the hydrophobic structures between β-sheets of amyloid oligomers make them resistant to enzymatic breakdown (65). Thus, we hypothesize that there are fewer Aβ_1-40_ inclusions embedded in the biofilm because Aβ_1-40_ exists as monomers and low-weight oligomers for a longer time than Aβ_1-42_, providing more chance for degradation by gingipains.

Proteolysis of antimicrobial peptides, including human peptides, is a key strategy of *P. gingivalis* to evade the host’s immune response (66, 67, 68). Interestingly, we observed that deletion of arginine-specific gingipains (RgpA/RgpB) but not of the lysine-specific gingipain (Kgp) negated Aβ degradation, despite lysine residues being present in both Aβ subtypes. These two types of gingipains exhibit differences in activity and inhibitory conditions, which may explain why only RgpA/RgpB appeared to degrade Aβ_40/42_ under our conditions (69, 70). Further experiments are required to determine if it is also the case *in situ*.

Aβ_1-42_ differs from Aβ_1-40_ only by the addition of an isoleucine and an alanine in the C-terminal, which increases Aβ_1-42_ overall aggregation rate into protofibrils and fibrils compared to Aβ_1-40_ (71, 72). These properties are likely sufficient to explain the difference observed in our study. When co-inoculated with *P. gingivalis*, Aβ_1-42_ auto-aggregates quickly into large, stable oligomers and fibrils, which reduce the likelihood of interacting with biofilm components and being degraded by the gingipains. These oligomers and fibrils would then integrate into the developing biofilm, promoting cell clumping and increasing overall matrix biomass. In contrast, Aβ_1-40_ remains in an equilibrium of monomers and low-complexity oligomers for a significant period. This period enables interaction between the biofilm matrix components and Aβ_1-40_, leading to alterations to their normal structure. Indeed, Aβ_1-40_ fibrils formed unusual bundles rather than long single fibers, and the biofilm formed dispersed clumps rather than covering most of the available surface.

*P. gingivalis* biofilm components were identified in Aβ plaques isolated from AD patients, suggesting that a co-aggregation phenomenon with Aβ_1-40_ and/or an integration of Aβ_1-42_ aggregates in the matrix could occur within the brain (44, 45). While the mechanisms underlying biofilm presence in the CNS are still poorly understood, outer membrane vesicles of *P. gingivalis* are found in the brains of AD patients and contain important biofilm components, such as FimA (53, 73–76). Modulation of Aβ aggregation through physical interaction with viral or bacterial proteins is a key factor that may explain how microorganisms would contribute to AD development (61, 77, 78). *P. gingivalis* biofilms are also routinely observed on teeth (79), and consequently, our observations demonstrate a potential interaction between *P. gingivalis* and Aβ outside of the CNS. Aggregates of Aβ were observed on the tooth surface and embedded within the oral biofilm of patients with AD (43), findings that are consistent with our observations. These non-CNS interactions are important, since peripheral infections of *P. gingivalis* were also linked to AD-like dementia in mice (41, 52).

The *P. gingivalis* extracellular matrix component that interacts with Aβ_1-40_ and alters its aggregation dynamics has yet to be identified. Also, FimA subtypes, capsular polysaccharides, and hemagglutinin expression vary between strains, which may influence biofilm effects on Aβ_40/42_ (80, 81). Nonetheless, by shedding light on the interaction between Aβ_40/42_ and *P. gingivalis*, our study offers a new perspective on this bacterium’s potential role in the onset of AD. Although the exact etiology remains unknown, a better understanding of the mechanisms underlying major risk factors, such as periodontitis, can offer valuable insights into AD prevention and treatment strategies.

## MATERIALS AND METHODS

### Aβ preparation

Aβ_1-40_ and Aβ_1-42_ (Genscript, NJ, USA) were prepared as previously described (82). Briefly, 1 mg of lyophilized Aβ_40/42_ was dissolved in a solution of NH_4_OH 10% (wt/vol) to 0.5 mg/mL. The peptides were incubated for 10 min at room temperature before being sonicated for 5 min and aliquoted in microcentrifuge tubes. The aliquots were then lyophilized overnight and stored at −80°C. While lyophilization should remove NH_4_OH, we also verified that it did not influence biofilm formation at the maximum working concentration (Fig. S4).

### Strain and media

*Porphyromonas gingivalis* ATCC 33277, KDP112, KDP128, and KDP129 were generously provided by Daniel Grenier (Université Laval, Québec). Strains KDP112 (∆*rgpA* ∆*rgpB*), KDP129 (∆*kgp*), and KDP128 (∆*rgpA* ∆*rgpB* ∆*kgp*) are gingipain deletion mutants in the *P. gingivalis* ATCC 33277 genetic background (83, 84). Cultures were routinely maintained in Tryptic Soy broth supplemented with hemin and vitamin K (TSBHK; 17 g/L casein peptone, 2.5 g/L K_2_HPO_4_, 2.5 g/L glucose, 5 g/L NaCl, 3 g/L soy peptone, 5 μg/mL hemin, and 1 μg/mL vitamin K) in a type-B vinyl COY anaerobic chamber with the following gas mix: 80% N_2_:10% CO_2_:10% H_2_, in static conditions. For biofilm-inducing conditions, *P. gingivalis* was cultured statically in TSBHK supplemented with Tryptone 1% (wt/vol) (TSBHKT) (85).

### Crystal violet assay

Crystal violet assays were used to assess biofilm production under different concentrations of Aβ_40/42_ (86). First, 36 h cultures of *P. gingivalis* were centrifuged at 3,200 × *g* for 20 min and resuspended in TSBHKT. Aβ aliquots were resuspended in 60 mM NaOH and diluted to the proper concentration in the same medium. Biofilm cultures were then performed in polystyrene 48-well plates containing 200 μL of TSBHKT inoculated with *P. gingivalis* at OD_600_ = 0.005 (5.625 × 10^6^ CFU/mL) and incubated for 24 h at 37°C. Of note, this concentration of *P. gingivalis* was selected after optimization for the most robust biofilm formation in our conditions. The remaining biofilm was washed with phosphate-buffered saline (PBS; 0.2 g/L KCl, 1.42 g/L Na_2_HPO_4_, and 0.24 g/L KH_2_PO_4_, 0.8 g/L NaCl, pH 7.4) before being covered with 200 μL of 0.01% (wt/vol) crystal violet for 20 min at room temperature. Each well was then washed with sterile deionized water to remove excess dye, resuspended in 33% (vol/vol) acetic acid, and quantified at OD_590_.

### Imaging of biofilm components

For confocal imaging of biofilm with Aβ_40/42_, *P. gingivalis* was inoculated at OD_600_ = 0.005 in 200 μL TSBHKT + 25 μg/mL Aβ in flat clear-bottom microscopy 96-well plates (Ibidi, Gräfelfing, Germany) for 24 h at 37°C. Supernatants from the cultures were discarded, and the remaining biofilms were washed with PBS. The biofilms were then fixed for 7 min in 200 μL of 4% (vol/vol) paraformaldehyde and then blocked with 200 μL of PBS + 2% (wt/vol) bovine serum albumin for 1 h at room temperature. For the staining of biofilm components under high Aβ concentrations (Fig. 2), we used Concanavalin A-Alexa647 (ConA) (1 h at room temperature, 50 μM; Thermo Fisher, USA), ThT (30 min at room temperature, 100 μM; Millipore Sigma, USA), and DAPI (30 min at room temperature, 1 μg/mL; Millipore Sigma, MA, USA). Pictures of the biofilms were taken using an Olympus FV3000 (Olympus, JP) confocal microscope at 20× magnification (PlanApo objective [20×/0.75 NA {numerical aperture}]) with the following laser settings: 405 nm (DAPI), 445 nm (ThT), and 640 nm (ConA). Laser intensity and sensor sensitivity were the same for all conditions. Image analysis used at least 25 images per condition, spread over three biological replicates. To observe Aβ aggregation within the biofilm (Fig. 3), we used Concanavalin A-Alexa647 (1 h at room temperature, 50 μM, Thermo Fisher, USA), 6C3 anti-amyloid beta peptide monoclonal antibody (1 h at room temperature, 1:1000, Millipore Sigma, MO, USA), Goat anti-Mouse IgG (H+L) Cross-Adsorbed Secondary Antibody, Alexa Fluor 555 (1 h at room temperature, 2 μg/mL, Thermo Fisher, USA), and DAPI (30 min at room temperature, 1 μg/mL, Thermo Fisher, USA). The images were produced with the same confocal microscope at 40× magnification (PlanApo objective [40×/0.95 NA]) with the following laser settings: 405 nm (DAPI), 561 nm (AlexaFluor 555), and 640 nm (ConA), and the analysis was performed using at least 25 images over three biological replicates. Laser intensity and sensor sensitivity were the same for all conditions.

### Microscopy analysis

For the fluorescence intensity measurements, Z-stack confocal images were initially preprocessed using Fiji. For each image, Z-sum intensity projection was applied, followed by a rolling ball background subtraction. Subsequently, whole-image intensity measurements were obtained using CellProfiler image analysis software. For the colocalization analysis, Fiji was used to perform maximum intensity projection on Z-stack images. Pixel colocalization was then measured on projection images using the Measure Colocalization module of CellProfiler 4.2.6 (87). Mander’s coefficient was used to measure the correlation between different fluorescence channels (88).

### Biofilm seeds preparation

Biofilm fragment preparation was performed as described elsewhere (77). Briefly, multiple wells in a 96-well plate were inoculated with *P. gingivalis* 33277 in 200 μL of TSBHKT at OD_600_ = 0.005. The biofilm was allowed to form under static conditions at 37°C for 24 h. The biofilm was then scooped in sterile PBS and vortexed at low intensity for 10 min with 25 μm glass beads to detach the cells from the extracellular matrix. It was then spun down at 3,200 × *g* for 10 min to remove most of the cells. The supernatant was then collected and quantified using Pierce BCA Protein Assay Kit (Thermo Scientific, MA, USA). Notably, proteomic analysis of this extracellular biofilm material confirmed the presence of all major and minor fimbrial subunits. CFU quantification revealed that for the same amount of proteinaceous material, there are 10^3^ fewer cells in this extracellular biofilm preparation than in the original biofilm (Fig. S5). This preparation was thereby named “seeds” and consists of a suspension of extracellular matrix components in PBS.

### Aggregation kinetics assays with ThT

For aggregation kinetics assays with ThT, Aβ aliquots were resuspended in 10 μL of 60 mM NaOH and incubated for 5 min at room temperature. They were then diluted to 10 μg/mL in PBS containing 30 μM ThT and 200 μL were distributed in black 96-well polystyrene plates (Corning, NY, USA) in the presence of 500 ng protein/mL of biofilm seeds. The assay wells were surrounded by two layers of wells filled with water to prevent evaporation. Plates were read every 5 min using a TECAN SPARK plate reader at 440 ± 20 nm excitation and 485 ± 20 nm emission, with constant agitation at 37°C. For each assay, a negative control containing only biofilm seeds and ThT was included to account for fluorescence from the seeds. These values remained flat throughout the assay and were subtracted from the fluorescence intensity measured in the experimental wells (Fig. S6). The resulting curves were analyzed to assess kinetic values as previously reported elsewhere (89). Briefly, the aggregation kinetics results were fitted to a sigmoidal curve (equation 1), where *k* is the apparent aggregation constant and *t*_1/2_ is the time to reach half-maximal fluorescence.


(1) $$Y=Y_{0}+\frac{Y_{max}-Y_{0}}{1+e^{-\left(t-t_{1/2}\right)k}}$$

From this equation, it is also possible to measure the lag phase using equation 2.


(2) $$\text{Lag}=t_{1/2}-\frac{2}{k}$$

### AFM

Experiments leading to AFM were performed in the same way as aggregation kinetics assays with ThT. Briefly, 100 μg/mL solutions of Aβ_40/42_ were incubated in PB buffer (0.2 g/L KCl, 1.42 g/L Na_2_HPO_4_, and 0.24 g/L KH_2_PO_4_, pH 7.4) with 5 μg protein/mL of biofilm fragments. Aβ_40/42_ samples were incubated in static conditions at 37°C for 3 h for Aβ_1-40_ and 1 h for Aβ_1-42_ before being adsorbed in a humid atmosphere on a clean mica surface (Electron Microscopy Sciences, PA, USA) for 30 min. After adsorption, mica slides were washed twice with ddH_2_O and dried under a nitrogen stream. AFM was performed using a Veeco Dimension Icon microscope (Bruker, USA) using ScanAsyst air probes (Bruker, resonance frequency 70 kHz, spring constant 0.4 N/m, tip nominal radius 2 nm). For each condition, 15 images were captured over three biological replicates for an area size of 10 μm × 10 μm.

### Gingipain assays

*P. gingivalis* WT and the gingipain mutants were statically grown in TSBHK for 48 h and adjusted to OD_600_ = 1. Cells were then removed by centrifugation at 3,200 × *g* for 20 min. Ten microliters of *P. gingivalis* supernatant was then mixed with 190 μL of a 5 μg/mL Aβ_40/42_ solution in PBS. Samples were incubated at 37°C for 16 h before being analyzed by dot blotting. Twenty microliters of sample was spotted onto a PVDF membrane and immunoblotted using MOAB-2 6C3 anti-amyloid beta peptide primary antibody (1 h at room temperature, 1:2,500, Sigma, MO, USA) and Peroxidase AffiniPure Goat Anti-Mouse IgG (H+L) (1 h at room temperature, 1:5,000; Jackson ImmunoResearch, PA, USA). Membranes were revealed using Clarity Max ECL Western Blotting Substrates (Bio-Rad, USA) and imaged through a Bio-Rad ChemiDoc using chemiluminescence. To observe the difference between non-aggregated and aggregated Aβ_40/42_, a sample of each Aβ subtype was initially incubated in PBS at 37°C for 24 h. This sample was compared to a fresh aliquot of Aβ and analyzed using the method described above. Densitometry analysis for each sample was performed using the integrated intensity values provided in ImageJ. Briefly, identical areas were overlaid on the dot blot images, and integrated intensity was measured for each dot.

### Statistical analysis

Statistical analyses were performed in GraphPad Prism 10. Comparisons were done using Student’s *t*-test, one-way ANOVA, Mann-Whitney or Kruskal-Wallis tests, all with 95% confidence intervals. Normality was assessed using Shapiro-Wilk’s normality test, and each result has been replicated in at least three independent biological replicates.

## SUPPLEMENTAL MATERIAL

Supplemental figures spectrum.01981-25-s0001.pdf Figures S1 to S6.

ASM does not own the copyrights to Supplemental Material that may be linked to, or accessed through, an article. The authors have granted ASM a non-exclusive, world-wide license to publish the Supplemental Material files. Please contact the corresponding author directly for reuse.

## References

[1] Slots J. 2000. Periodontitis: facts, fallacies and the future. Periodontol 75:7–23. doi:10.1111/prd.12221 28758294

[2] Mohanty R, Asopa SJ, Joseph MD, Singh B, Rajguru JP, Saidath K, Sharma U. 2019. Red complex: polymicrobial conglomerate in oral flora: a review. J Family Med Prim Care 8:3480–3486. doi:10.4103/jfmpc.jfmpc_759_19 31803640 PMC6881954

[3] Mysak J, Podzimek S, Sommerova P, Lyuya-Mi Y, Bartova J, Janatova T, Prochazkova J, Duskova J. 2014. Porphyromonas gingivalis: major periodontopathic pathogen overview. J Immunol Res 2014:476068. doi:10.1155/2014/476068 24741603 PMC3984870

[4] Sakanaka A, Takeuchi H, Kuboniwa M, Amano A. 2016. Dual lifestyle of Porphyromonas gingivalis in biofilm and gingival cells. Microb Pathog 94:42–47. doi:10.1016/j.micpath.2015.10.003 26456558

[5] Olsen I, Lambris JD, Hajishengallis G. 2017. Porphyromonas gingivalis disturbs host-commensal homeostasis by changing complement function. J Oral Microbiol 9:1340085. doi:10.1080/20002297.2017.1340085 28748042 PMC5508361

[6] Carlisle MD, Srikantha RN, Brogden KA. 2009. Degradation of human alpha- and beta-defensins by culture supernatants of Porphyromonas gingivalis strain 381. J Innate Immun 1:118–122. doi:10.1159/000181015 20375570 PMC7312840

[7] Curtis MA, Kuramitsu HK, Lantz M, Macrina FL, Nakayama K, Potempa J, Reynolds EC, Aduse-Opoku J. 1999. Molecular genetics and nomenclature of proteases of Porphyromonas gingivalis. J Periodontal Res 34:464–472. doi:10.1111/j.1600-0765.1999.tb02282.x 10697803

[8] Katz J, Yang Q-B, Zhang P, Potempa J, Travis J, Michalek SM, Balkovetz DF. 2002. Hydrolysis of epithelial junctional proteins by Porphyromonas gingivalis gingipains. Infect Immun 70:2512–2518. doi:10.1128/IAI.70.5.2512-2518.2002 11953390 PMC127922

[9] Popadiak K, Potempa J, Riesbeck K, Blom AM. 2007. Biphasic effect of gingipains from Porphyromonas gingivalis on the human complement system. J Immunol 178:7242–7250. doi:10.4049/jimmunol.178.11.7242 17513773

[10] Takii R, Kadowaki T, Baba A, Tsukuba T, Yamamoto K. 2005. A functional virulence complex composed of gingipains, adhesins, and lipopolysaccharide shows high affinity to host cells and matrix proteins and escapes recognition by host immune systems. Infect Immun 73:883–893. doi:10.1128/IAI.73.2.883-893.2005 15664930 PMC547079

[11] Lamont RJ, Jenkinson HF. 2000. Subgingival colonization by Porphyromonas gingivalis. Oral Microbiol Immunol 15:341–349. doi:10.1034/j.1399-302x.2000.150601.x 11154429

[12] Xu W, Zhou W, Wang H, Liang S. 2020. Roles of Porphyromonas gingivalis and its virulence factors in periodontitis. Adv Protein Chem Struct Biol 120:45–84. doi:10.1016/bs.apcsb.2019.12.001 32085888 PMC8204362

[13] Kuboniwa M, Amano A, Hashino E, Yamamoto Y, Inaba H, Hamada N, Nakayama K, Tribble GD, Lamont RJ, Shizukuishi S. 2009. Distinct roles of long/short fimbriae and gingipains in homotypic biofilm development by Porphyromonas gingivalis. BMC Microbiol 9:105. doi:10.1186/1471-2180-9-105 19470157 PMC2697998

[14] Gerits E, Verstraeten N, Michiels J. 2017. New approaches to combat Porphyromonas gingivalis biofilms. J Oral Microbiol 9:1300366. doi:10.1080/20002297.2017.1300366 28473880 PMC5405727

[15] Ali Mohammed MM, Nerland AH, Al-Haroni M, Bakken V. 2013. Characterization of extracellular polymeric matrix, and treatment of Fusobacterium nucleatum and Porphyromonas gingivalis biofilms with DNase I and proteinase K. J Oral Microbiol 5:20015. doi:10.3402/jom.v5i0.20015 PMC3559756 23372876

[16] Ishikawa M, Yoshida K, Okamura H, Ochiai K, Takamura H, Fujiwara N, Ozaki K. 2013. Oral Porphyromonas gingivalis translocates to the liver and regulates hepatic glycogen synthesis through the Akt/GSK-3β signaling pathway. Biochim Biophys Acta Mol Basis Dis 1832:2035–2043. doi:10.1016/j.bbadis.2013.07.012 23899607

[17] Mulhall H, Huck O, Amar S. 2020. Porphyromonas gingivalis, a long-range pathogen: systemic impact and therapeutic implications. Microorganisms 8:1–15. doi:10.3390/microorganisms8060869 PMC7357039 32526864

[18] Velsko IM, Chukkapalli SS, Rivera MF, Lee J-Y, Chen H, Zheng D, Bhattacharyya I, Gangula PR, Lucas AR, Kesavalu L. 2014. Active invasion of oral and aortic tissues by Porphyromonas gingivalis in mice causally links periodontitis and atherosclerosis. PLoS One 9:e97811. doi:10.1371/journal.pone.0097811 24836175 PMC4024021

[19] Detert J, Pischon N, Burmester GR, Buttgereit F. 2010. The association between rheumatoid arthritis and periodontal disease. Arthritis Res Ther 12:218. doi:10.1186/ar3106 21062513 PMC2990988

[20] Chopra A, Radhakrishnan R, Sharma M. 2020. Porphyromonas gingivalis and adverse pregnancy outcomes: a review on its intricate pathogenic mechanisms. Crit Rev Microbiol 46:213–236. doi:10.1080/1040841X.2020.1747392 32267781

[21] Kanagasingam S, Chukkapalli SS, Welbury R, Singhrao SK. 2020. Porphyromonas gingivalis is a strong risk factor for Alzheimer’s disease. J Alzheimers Dis Rep 4:501–511. doi:10.3233/ADR-200250 33532698 PMC7835991

[22] Querfurth HW, LaFerla FM. 2010. Alzheimer’s disease. N Engl J Med 362:329–344. doi:10.1056/NEJMra0909142 20107219

[23] Arispe N, Diaz JC, Simakova O. 2007. Aβ ion channels. prospects for treating Alzheimer’s disease with Aβ channel blockers. Biochim Biophys Acta Biomembr 1768:1952–1965. doi:10.1016/j.bbamem.2007.03.014 17490607

[24] Arispe N, Rojas E, Pollard HB. 1993. Alzheimer disease amyloid beta protein forms calcium channels in bilayer membranes: blockade by tromethamine and aluminum. Proc Natl Acad Sci USA 90:567–571. doi:10.1073/pnas.90.2.567 8380642 PMC45704

[25] Lustbader JW, Cirilli M, Lin C, Xu HW, Takuma K, Wang N, Caspersen C, Chen X, Pollak S, Chaney M, Trinchese F, Liu S, Gunn-Moore F, Lue L-F, Walker DG, Kuppusamy P, Zewier ZL, Arancio O, Stern D, Yan SS, Wu H. 2004. ABAD directly links Aß to mitochondrial toxicity in Alzheimer’s disease. Science 304:448–452. doi:10.1126/science.1091230 15087549

[26] Jung ES, Suh K, Han J, Kim H, Kang H-S, Choi W-S, Mook-Jung I. 2022. Amyloid-β activates NLRP3 inflammasomes by affecting microglial immunometabolism through the Syk-AMPK pathway. Aging Cell 21:e13623. doi:10.1111/acel.13623 35474599 PMC9124305

[27] Gu L, Guo Z. 2013. Alzheimer’s Aβ42 and Aβ40 peptides form interlaced amyloid fibrils. J Neurochem 126:305–311. doi:10.1111/jnc.12202 23406382 PMC3716832

[28] Kuo YM, Kokjohn TA, Watson MD, Woods AS, Cotter RJ, Sue LI, Kalback WM, Emmerling MR, Beach TG, Roher AE. 2000. Elevated abeta42 in skeletal muscle of Alzheimer disease patients suggests peripheral alterations of AbetaPP metabolism. Am J Pathol 156:797–805. doi:10.1016/s0002-9440(10)64947-4 10702395 PMC1876838

[29] Roher AE, Esh CL, Kokjohn TA, Castaño EM, Van Vickle GD, Kalback WM, Patton RL, Luehrs DC, Daugs ID, Kuo Y-M, Emmerling MR, Soares H, Quinn JF, Kaye J, Connor DJ, Silverberg NB, Adler CH, Seward JD, Beach TG, Sabbagh MN. 2009. Amyloid beta peptides in human plasma and tissues and their significance for Alzheimer’s disease. Alzheimers Dement 5:18–29. doi:10.1016/j.jalz.2008.10.004 19118806 PMC2663406

[30] Whitson HE, Colton C, El Khoury J, Gate D, Goate A, Heneka MT, Kaddurah-Daouk R, Klein RS, Shinohara ML, Sisodia S, Spudich SS, Stevens B, Tanzi R, Ting JP, Garden G, Aiello A, Chiba-Falek O, Heitman J, Johnson KG, Luftig M, Moseman A, Rawls J, Shinohara ML, Swanstrom R, Terrando N. 2022. Infection and inflammation: new perspectives on Alzheimer’s disease. Brain, Behavior, & Immunity - Health 22:100462. doi:10.1016/j.bbih.2022.100462 PMC9475126 36118272

[31] Soscia SJ, Kirby JE, Washicosky KJ, Tucker SM, Ingelsson M, Hyman B, Burton MA, Goldstein LE, Duong S, Tanzi RE, Moir RD. 2010. The Alzheimer’s disease-associated amyloid β-protein is an antimicrobial peptide. PLoS One 5:e9505. doi:10.1371/journal.pone.0009505 20209079 PMC2831066

[32] Moir RD, Lathe R, Tanzi RE. 2018. The antimicrobial protection hypothesis of Alzheimer’s disease. Alzheimers Dement 14:1602–1614. doi:10.1016/j.jalz.2018.06.3040 30314800

[33] Bourgade K, Le Page A, Bocti C, Witkowski JM, Dupuis G, Frost EH, Fülöp T. 2016. Protective effect of amyloid-β peptides against herpes simplex virus-1 infection in a neuronal cell culture model. J Alzheimers Dis 50:1227–1241. doi:10.3233/JAD-150652 26836158

[34] Pastore A, Raimondi F, Rajendran L, Temussi PA. 2020. Why does the Aβ peptide of Alzheimer share structural similarity with antimicrobial peptides? Commun Biol 3:135. doi:10.1038/s42003-020-0865-9 32193491 PMC7081199

[35] Spitzer P, Condic M, Herrmann M, Oberstein TJ, Scharin-Mehlmann M, Gilbert DF, Friedrich O, Grömer T, Kornhuber J, Lang R, Maler JM. 2016. Amyloidogenic amyloid-β-peptide variants induce microbial agglutination and exert antimicrobial activity. Sci Rep 6:32228. doi:10.1038/srep32228 27624303 PMC5021948

[36] Villemagne VL, Burnham S, Bourgeat P, Brown B, Ellis KA, Salvado O, Szoeke C, Macaulay SL, Martins R, Maruff P, Ames D, Rowe CC, Masters CL, Australian Imaging Biomarkers and Lifestyle (AIBL) Research Group. 2013. Amyloid β deposition, neurodegeneration, and cognitive decline in sporadic Alzheimer’s disease: a prospective cohort study. Lancet Neurol 12:357–367. doi:10.1016/S1474-4422(13)70044-9 23477989

[37] Liu S, Butler CA, Ayton S, Reynolds EC, Dashper SG. 2024. Porphyromonas gingivalis and the pathogenesis of Alzheimer’s disease. Crit Rev Microbiol 50:127–137. doi:10.1080/1040841X.2022.2163613 36597758

[38] Arastu-Kapur S, Nguyen M, Raha D, Ermini F, Haditsch U, Araujo J, De Lannoy IAM, Ryder MI, Dominy SS, Lynch C, Holsinger LJ. 2020. Treatment of Porphyromonas gulae infection and downstream pathology in the aged dog by lysine-gingipain inhibitor COR388. Pharmacol Res Perspect 8:e00562. doi:10.1002/prp2.562 31999052 PMC6990966

[39] Dominy SS, Lynch C, Ermini F, Benedyk M, Marczyk A, Konradi A, Nguyen M, Haditsch U, Raha D, Griffin C, et al.. 2019. Porphyromonas gingivalis in Alzheimer’s disease brains: evidence for disease causation and treatment with small-molecule inhibitors. Sci Adv 5:eaau3333. doi:10.1126/sciadv.aau3333 30746447 PMC6357742

[40] Ilievski V, Zuchowska PK, Green SJ, Toth PT, Ragozzino ME, Le K, Aljewari HW, O’Brien-Simpson NM, Reynolds EC, Watanabe K. 2018. Chronic oral application of a periodontal pathogen results in brain inflammation, neurodegeneration and amyloid beta production in wild type mice. PLoS One 13:e0204941. doi:10.1371/journal.pone.0204941 30281647 PMC6169940

[41] Wu Z, Ni J, Liu Y, Teeling JL, Takayama F, Collcutt A, Ibbett P, Nakanishi H. 2017. Cathepsin B plays a critical role in inducing Alzheimer’s disease-like phenotypes following chronic systemic exposure to lipopolysaccharide from Porphyromonas gingivalis in mice. Brain Behav Immun 65:350–361. doi:10.1016/j.bbi.2017.06.002 28610747

[42] Liao Y, Chen H-W, Qiu C, Shen H, He Z-Y, Song Z-C, Zhou W. 2025. The detection of amyloid-β peptides in gingival crevicular fluid and its inuence on oral pathogens. Mol Oral Microbiol 40:94–103. doi:10.1111/omi.12488 39668581

[43] Kanagasingam S, von Ruhland C, Welbury R, Singhrao SK. 2022. Ex vivo detection of amyloid-β in naturally formed oral biofilm. J Alzheimers Dis Rep 6:757–773. doi:10.3233/ADR-220076 36721488 PMC9837734

[44] Allen HB. 2016. Alzheimer’s disease: assessing the role of spirochetes, biofilms, the immune system, and amyloid-β with regard to potential treatment and prevention. J Alzheimers Dis 53:1271–1276. doi:10.3233/JAD-160388 27372648 PMC5008232

[45] Miklossy J. 2016. Bacterial amyloid and DNA are important constituents of senile plaques: Further evidence of the spirochetal and biofilm nature of senile plaques, p 1459–1473. In Handbook of Infection and Alzheimer’s Disease. Vol. 53. 10.3233/JAD-160451 PMC4981904 27314530

[46] Mirzaei R, Mohammadzadeh R, Sholeh M, Karampoor S, Abdi M, Dogan E, Moghadam MS, Kazemi S, Jalalifar S, Dalir A, Yousefimashouf R, Mirzaei E, Khodavirdipour A, Alikhani MY. 2020. The importance of intracellular bacterial biofilm in infectious diseases. Microb Pathog 147:104393. doi:10.1016/j.micpath.2020.104393 32711113

[47] Gade Malmos K, Blancas-Mejia LM, Weber B, Buchner J, Ramirez-Alvarado M, Naiki H, Otzen D. 2017. ThT 101: a primer on the use of thioflavin T to investigate amyloid formation. Amyloid 24:1–16. doi:10.1080/13506129.2017.1304905 28393556

[48] Zeriouh H, de Vicente A, Pérez-García A, Romero D. 2014. Surfactin triggers biofilm formation of Bacillus subtilis in melon phylloplane and contributes to the biocontrol activity. Environ Microbiol 16:2196–2211. doi:10.1111/1462-2920.12271 24308294

[49] Li N, Collyer CA. 2011. Gingipains from Porphyromonas gingivalis - Complex domain structures confer diverse functions. Eur J Microbiol Immunol (Bp) 1:41–58. doi:10.1556/EuJMI.1.2011.1.7 24466435 PMC3894813

[50] Yoon SS, Jo SA. 2012. Mechanisms of amyloid-β peptide clearance: potential therapeutic targets for Alzheimer’s disease. Biomol Ther (Seoul) 20:245–255. doi:10.4062/biomolther.2012.20.3.245 24130920 PMC3794520

[51] Nie R, Wu Z, Ni J, Zeng F, Yu W, Zhang Y, Kadowaki T, Kashiwazaki H, Teeling JL, Zhou Y. 2019 a. Porphyromonas gingivalis infection induces amyloid-β accumulation in monocytes/macrophages. J Alzheimers Dis 72:479–494. doi:10.3233/JAD-190298 31594220

[52] Nie R, Wu Z, Ni J, Zeng F, Yu W, Zhang Y, Kadowaki T, Kashiwazaki H, Teeling JL, Zhou Y. 2019. Porphyromonas gingivalis infection induces amyloid-β accumulation in monocytes/macrophages. JAD 72:479–494. doi:10.3233/JAD-190298 31594220

[53] Singhrao SK, Olsen I. 2018. Are Porphyromonas gingivalis outer membrane vesicles microbullets for sporadic Alzheimer’s disease manifestation? J Alzheimers Dis Rep 2:219–228. doi:10.3233/ADR-180080 30599043 PMC6311351

[54] Singhrao SK, Olsen I. 2019. Assessing the role of Porphyromonas gingivalis in periodontitis to determine a causative relationship with Alzheimer’s disease. J Oral Microbiol 11:1563405. doi:10.1080/20002297.2018.1563405 30728914 PMC6352933

[55] Díaz-Zúñiga J, More J, Melgar-Rodríguez S, Jiménez-Unión M, Villalobos-Orchard F, Muñoz-Manríquez C, Monasterio G, Valdés JL, Vernal R, Paula-Lima A. 2020. Alzheimer’s disease-like pathology triggered by Porphyromonas gingivalis in wild type rats is serotype dependent. Front Immunol 11:588036. doi:10.3389/fimmu.2020.588036 33240277 PMC7680957

[56] Aravindraja C, Sakthivel R, Liu X, Goodwin M, Veena P, Godovikova V, Fenno JC, Levites Y, Golde TE, Kesavalu L. 2022. Intracerebral but not peripheral infection of live Porphyromonas gingivalis exacerbates Alzheimer’s disease like amyloid pathology in APP-TgCRND8 mice. Int J Mol Sci 23:3328. doi:10.3390/ijms23063328 35328748 PMC8954230

[57] Kanagasingam S, von Ruhland C, Welbury R, Chukkapalli SS, Singhrao SK. 2022. Porphyromonas gingivalis conditioned medium induces amyloidogenic processing of the amyloid-β protein precursor upon in vitro infection of SH-SY5Y cells. J Alzheimers Dis Rep 6:577–587. doi:10.3233/ADR-220029 36275415 PMC9535609

[58] Chen G-F, Xu T-H, Yan Y, Zhou Y-R, Jiang Y, Melcher K, Xu HE. 2017. Amyloid beta: structure, biology and structure-based therapeutic development. Acta Pharmacol Sin 38:1205–1235. doi:10.1038/aps.2017.28 28713158 PMC5589967

[59] Dueholm MS, Søndergaard MT, Nilsson M, Christiansen G, Overgaard MT, Nielsen PH. 2013. Expression of Fap amyloids in Pseudomonas aeruginosa, P. fluorescens, and P. putida results in aggregation and increased biofilm formation. Microbiology Open 2:365–382. doi:10.1002/mbo3.81 23504942 PMC3684753

[60] Kumar DKV, Choi SH, Washicosky KJ, Eimer WA, Tucker S, Ghofrani J, Lefkowitz A, McColl G, Goldstein LE, Tanzi RE, Moir RD. 2016. Amyloid-β peptide protects against microbial infection in mouse and worm models of Alzheimer’s disease. Sci Transl Med 8:340ra72. doi:10.1126/scitranslmed.aaf1059 PMC5505565 27225182

[61] Ali SA, Chung KHK, Forgham H, Olsen WP, Kakinen A, Balaji A, Otzen DE, Davis TP, Javed I. 2023 a. Alzheimer’s progenitor amyloid‐β targets and dissolves microbial amyloids and impairs biofilm function. Adv Sci (Weinh) 10. doi:10.1002/advs.202301423 PMC10582422 37594661

[62] Prosswimmer T, Heng A, Daggett V. 2024. Mechanistic insights into the role of amyloid-β in innate immunity. Sci Rep 14. doi:10.1038/s41598-024-55423-9 PMC10912764 38438446

[63] Ren B, Zhang Y, Zhang M, Liu Y, Zhang D, Gong X, Feng Z, Tang J, Chang Y, Zheng J. 2019. Fundamentals of cross-seeding of amyloid proteins: an introduction. J Mater Chem B 7:7267–7282. doi:10.1039/C9TB01871A 31647489

[64] Karygianni L, Ren Z, Koo H, Thurnheer T. 2020. Biofilm matrixome: extracellular components in structured microbial communities. Trends Microbiol 28:668–681. doi:10.1016/j.tim.2020.03.016 32663461

[65] Eisenberg D, Jucker M. 2012. The amyloid state of proteins in human diseases. Cell 148:1188–1203. doi:10.1016/j.cell.2012.02.022 22424229 PMC3353745

[66] Gutner M, Chaushu S, Balter D, Bachrach G. 2009. Saliva enables the antimicrobial activity of LL-37 in the presence of proteases of Porphyromonas gingivalis. Infect Immun 77:5558–5563. doi:10.1128/IAI.00648-09 19805540 PMC2786438

[67] Maisetta G, Brancatisano FL, Esin S, Campa M, Batoni G. 2011. Gingipains produced by Porphyromonas gingivalis ATCC49417 degrade human-β-defensin 3 and affect peptide’s antibacterial activity in vitro. Peptides 32:1073–1077. doi:10.1016/j.peptides.2011.02.003 21335044

[68] Tada H, Sugawara S, Nemoto E, Takahashi N, Imamura T, Potempa J, Travis J, Shimauchi H, Takada H. 2002. Proteolysis of CD14 on human gingival fibroblasts by arginine-specific cysteine proteinases from Porphyromonas gingivalis leading to down-regulation of lipopolysaccharide-induced interleukin-8 production. Infect Immun 70:3304–3307. doi:10.1128/IAI.70.6.3304-3307.2002 12011031 PMC127988

[69] Tancharoen S, Matsuyama T, Kawahara K-I, Tanaka K, Lee L-J, Machigashira M, Noguchi K, Ito T, Imamura T, Potempa J, Kikuchi K, Maruyama I. 2015. Cleavage of host cytokeratin-6 by lysine-specific gingipain induces gingival inflammation in periodontitis patients. PLoS One 10:e0117775. doi:10.1371/journal.pone.0117775 25688865 PMC4331500

[70] Fujimura S, Hirai K, Shibata Y, Nakayama K, Nakamura T. 1998. Comparative properties of envelope-associated arginine-gingipains and lysine-gingipain of Porphyromonas gingivalis. FEMS Microbiol Lett 163:173–179. doi:10.1111/j.1574-6968.1998.tb13042.x 9673019

[71] Hampel H, Hardy J, Blennow K, Chen C, Perry G, Kim SH, Villemagne VL, Aisen P, Vendruscolo M, Iwatsubo T, Masters CL, Cho M, Lannfelt L, Cummings JL, Vergallo A. 2021. The amyloid-β pathway in Alzheimer’s disease. Mol Psychiatry 26:5481–5503. doi:10.1038/s41380-021-01249-0 34456336 PMC8758495

[72] Qiu T, Liu Q, Chen YX, Zhao YF, Li YM. 2015. Aβ42 and Aβ40: similarities and differences. J Pept Sci 21:522–529. doi:10.1002/psc.2789 26018760

[73] Okamura H, Hirota K, Yoshida K, Weng Y, He Y, Shiotsu N, Ikegame M, Uchida-Fukuhara Y, Tanai A, Guo J. 2021. Outer membrane vesicles of Porphyromonas gingivalis: novel communication tool and strategy. Jpn Dent Sci Rev 57:138–146. doi:10.1016/j.jdsr.2021.07.003 34484474 PMC8399048

[74] Gui MJ, Dashper SG, Slakeski N, Chen YY, Reynolds EC. 2016. Spheres of influence: Porphyromonas gingivalis outer membrane vesicles. Mol Oral Microbiol 31:365–378. doi:10.1111/omi.12134 26466922

[75] Qiu Y, Zhao Y, He G, Yang D. 2025. Porphyromonas gingivalis and its outer membrane vesicles induce neuroinflammation in mice through distinct mechanisms. Immunity Inflam & Disease 13. doi:10.1002/iid3.70135 PMC11811961 39932228

[76] Gong T, Chen Q, Mao H, Zhang Y, Ren H, Xu M, Chen H, Yang D. 2022. Outer membrane vesicles of Porphyromonas gingivalis trigger NLRP3 inflammasome and induce neuroinflammation, tau phosphorylation, and memory dysfunction in mice. Front Cell Infect Microbiol 12. doi:10.3389/fcimb.2022.925435 PMC9397999 36017373

[77] Javed I, Zhang Z, Adamcik J, Andrikopoulos N, Li Y, Otzen DE, Lin S, Mezzenga R, Davis TP, Ding F, Ke PC. 2020. Accelerated amyloid beta pathogenesis by bacterial amyloid FapC. Adv Sci (Weinh) 7:2001299. doi:10.1002/advs.202001299 32999841 PMC7509637

[78] Ezzat K, Pernemalm M, Pålsson S, Roberts TC, Järver P, Dondalska A, Bestas B, Sobkowiak MJ, Levänen B, Sköld M, et al.. 2019. The viral protein corona directs viral pathogenesis and amyloid aggregation. Nat Commun 10:2331. doi:10.1038/s41467-019-10192-2 31133680 PMC6536551

[79] Griffen AL, Becker MR, Lyons SR, Moeschberger ML, Leys EJ. 1998. Prevalence of Porphyromonas gingivalis and periodontal health status. J Clin Microbiol 36:3239–3242. doi:10.1128/JCM.36.11.3239-3242.1998 9774572 PMC105308

[80] Mendez KN, Hoare A, Soto C, Bugueño I, Olivera M, Meneses C, Pérez-Donoso JM, Castro-Nallar E, Bravo D. 2019. Variability in genomic and virulent properties of Porphyromonas gingivalis strains isolated from healthy and severe chronic periodontitis individuals. Front Cell Infect Microbiol 9:246. doi:10.3389/fcimb.2019.00246 31355151 PMC6635597

[81] Hasegawa Y, Nagano K. 2021. Porphyromonas gingivalis FimA and Mfa1 fimbriae: current insights on localization, function, biogenesis, and genotype. Jpn Dent Sci Rev 57:190–200. doi:10.1016/j.jdsr.2021.09.003 34691295 PMC8512630

[82] Ryan TM, Caine J, Mertens HDT, Kirby N, Nigro J, Breheney K, Waddington LJ, Streltsov VA, Curtain C, Masters CL, Roberts BR. 2013. Ammonium hydroxide treatment of Aβ produces an aggregate free solution suitable for biophysical and cell culture characterization. PeerJ 1:e73. doi:10.7717/peerj.73 23678397 PMC3646356

[83] Nakayama K, Kadowaki T, Okamoto K, Yamamoto K. 1995. Construction and characterization of arginine-specific cysteine proteinase (Arg-gingipain)-deficient mutants of Porphyromonas gingivalis. evidence for significant contribution of Arg-gingipain to virulence. J Biol Chem 270:23619–23626. doi:10.1074/jbc.270.40.23619 7559528

[84] Shi Y, Ratnayake DB, Okamoto K, Abe N, Yamamoto K, Nakayama K. 1999. Genetic analyses of proteolysis, hemoglobin binding, and hemagglutination of Porphyromonas gingivalis. Journal of Biological Chemistry 274:17955–17960. doi:10.1074/jbc.274.25.17955 10364243

[85] Davey ME. 2006. Techniques for the growth of Porphyromonas gingivalis biofilms. Periodontol 2000 42:27–35. doi:10.1111/j.1600-0757.2006.00183.x 16930304

[86] O’Toole GA, Kolter R. 1998. Initiation of biofilm formation in Pseudomonas fluorescens WCS365 proceeds via multiple, convergent signalling pathways: a genetic analysis. Mol Microbiol 28:449–461. doi:10.1046/j.1365-2958.1998.00797.x 9632250

[87] Lamprecht MR, Sabatini DM, Carpenter AE. 2007. CellProfiler: free, versatile software for automated biological image analysis. Biotechniques 42:71–75. doi:10.2144/000112257 17269487

[88] Manders EM, Stap J, Brakenhoff GJ, van Driel R, Aten JA. 1992. Dynamics of three-dimensional replication patterns during the S-phase, analysed by double labelling of DNA and confocal microscopy. J Cell Sci 103 (Pt 3):857–862. doi:10.1242/jcs.103.3.857 1478975

[89] Cabaleiro-Lago C, Quinlan-Pluck F, Lynch I, Lindman S, Minogue AM, Thulin E, Walsh DM, Dawson KA, Linse S. 2008. Inhibition of amyloid β protein fibrillation by polymeric nanoparticles. J Am Chem Soc 130:15437–15443. doi:10.1021/ja8041806 18954050

