# Supplementary material for: Bidirectional relationship between the biofilm of Porphyromonas gingivalis and the amyloid-beta peptide
Source: Microbiol Spectr. 2026 Jan 16;14(3):e01981-25. doi: 10.1128/spectrum.01981-25 (PMC12955484; doi:10.1128/spectrum.01981-25)
Supplement: Supplemental figures — Figures S1 to S6. [file spectrum.01981-25-s0001.pdf]

<u>**Supplementary Material**</u>

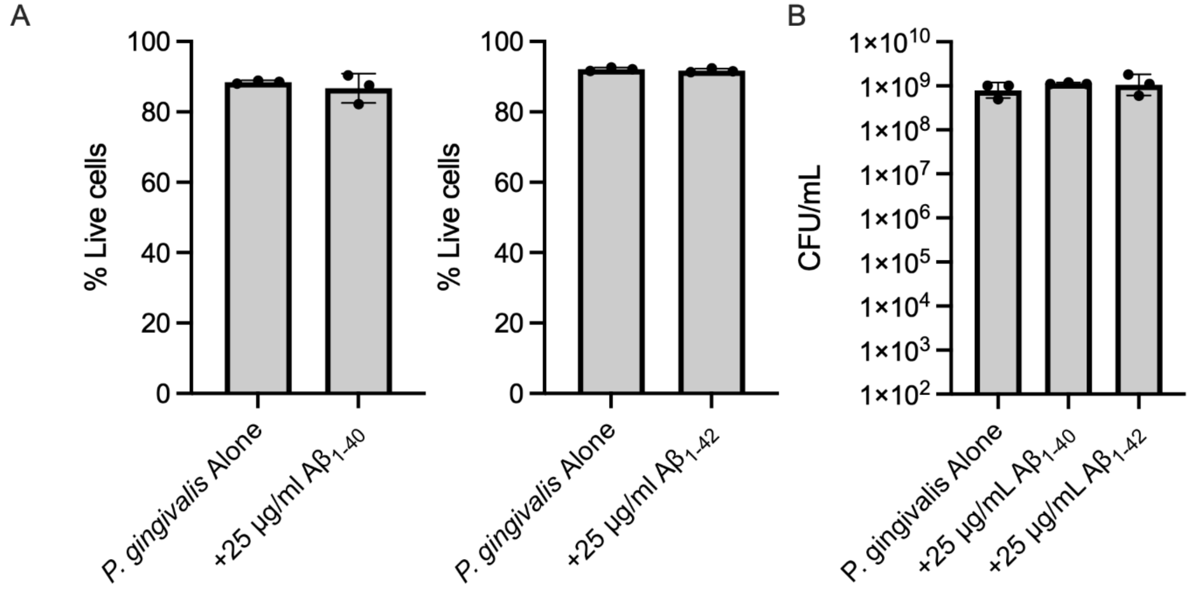

Figure S1 : (A) Percentage of live planktonic phase *Porphyromonas gingivalis* 33277 incubated 3 h with 25 µg/mL $A\beta_{1-40}$ (Left) or $A\beta_{1-42}$ (Right). Assessed using LIVE/DEAD staining in flow cytometry. Statistical analysis was performed using Mann-Whitney test for $A\beta_{1-40}$ and Student's T-test for $A\beta_{1-42}$. (B) Colony forming unit counts of *P. gingivalis* incubated 3 h with 25 µg/mL $A\beta_{1-40}$ or $A\beta_{1-42}$. Statistical analysis was done using Kruskal-Wallis with a Dunn's multiple comparisons test.

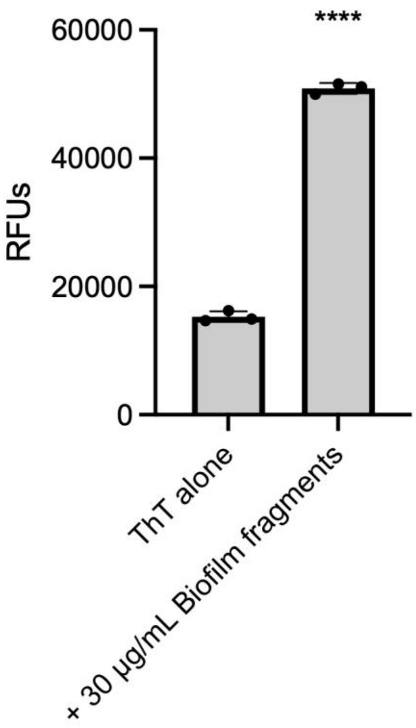

Figure S2: Thioflavin T staining of *Porphyromonas gingivalis* 33277 biofilm fragments. Statistical analysis was performed using Student’s T test. ****(P<0.0001).

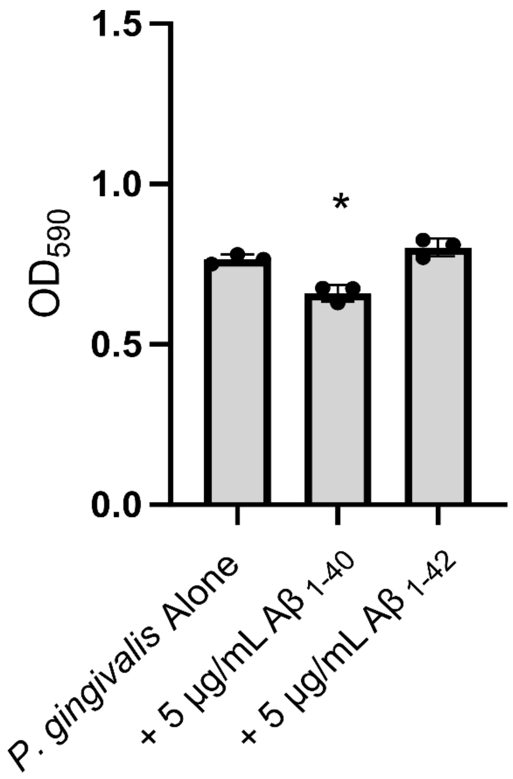

Figure S3: Effect of 5 μg/mL $A\beta_{1-40}$ or $A\beta_{1-42}$ on the biofilm of *Porphyromonas gingivalis* 33277. Statistical analysis was performed using Kruskal-Wallis with a Dunn's multiple comparisons test. *($P \leq 0.05$).

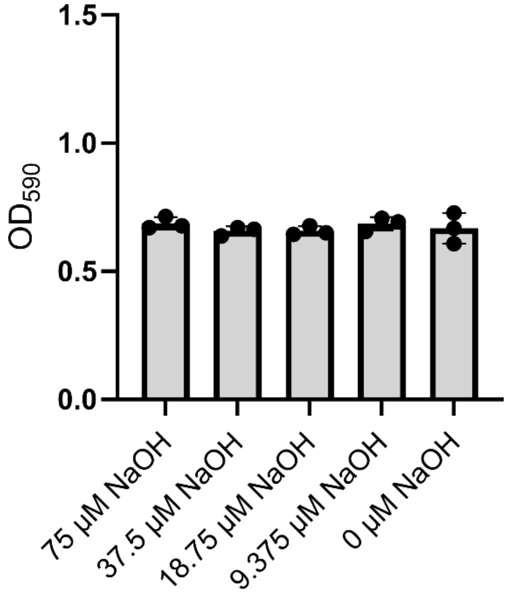

Figure S4 : Effect of NaOH on the biofilm of *Porphyromonas gingivalis* 33277. Concentrations correspond to the actual NaOH concentration in Figure 1. Statistical analysis was done using an ANOVA with a Dunnett's T3 multiple comparison test.

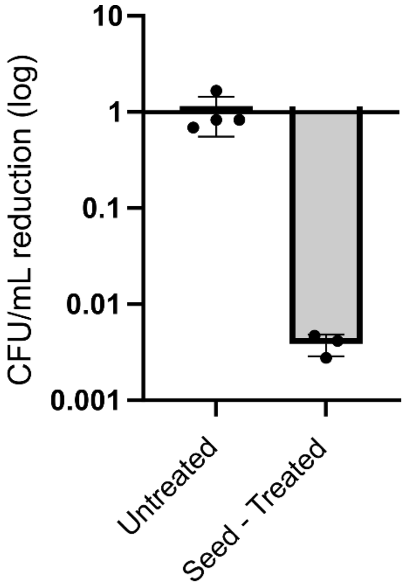

Figure S5 : Colony forming units reduction of *Porphyromonas gingivalis* 33277 following seed preparation.

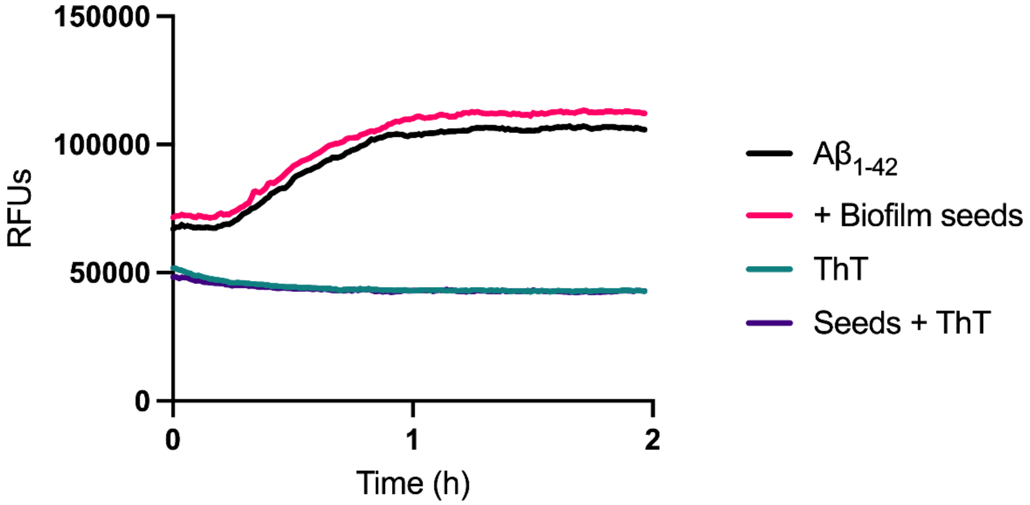

Figure S6: Fluorescence exhibited by biofilm seeds incubated alone with ThT compared to $A\beta_{1-42}$ amplification.
